# Learning-based sound speed estimation and aberration correction for linear-array photoacoustic imaging

**DOI:** 10.1016/j.pacs.2024.100621

**Published:** 2024-05-28

**Authors:** Mengjie Shi, Tom Vercauteren, Wenfeng Xia

**Affiliations:** School of Biomedical Engineering and Imaging Sciences, King’s College London, London, SE1 7EH, United Kingdom

**Keywords:** Photoacoustic imaging, Ultrasound imaging, Deep learning, Speed of sound estimation, Image reconstruction, Aberration correction

## Abstract

Photoacoustic (PA) image reconstruction involves acoustic inversion that necessitates the specification of the speed of sound (SoS) within the medium of propagation. Due to the lack of information on the spatial distribution of the SoS within heterogeneous soft tissue, a homogeneous SoS distribution (such as 1540 m/s) is typically assumed in PA image reconstruction, similar to that of ultrasound (US) imaging. Failure to compensate for the SoS variations leads to aberration artefacts, deteriorating the image quality. Various methods have been proposed to address this issue, but they usually involve complex hardware and/or time-consuming algorithms, hindering clinical translation. In this work, we introduce a deep learning framework for SoS estimation and subsequent aberration correction in a dual-modal PA/US imaging system exploiting a clinical US probe. As the acquired PA and US images were inherently co-registered, the estimated SoS distribution from US channel data using a deep neural network was incorporated for accurate PA image reconstruction. The framework comprised an initial pre-training stage based on digital phantoms, which was further enhanced through transfer learning using physical phantom data and associated SoS maps obtained from measurements. This framework achieved a root mean square error of 10.2 m/s and 15.2 m/s for SoS estimation on digital and physical phantoms, respectively and structural similarity index measures of up to 0.88 for PA reconstructions compared to the conventional approach of 0.69. A maximum of 1.2 times improvement in the signal-to-noise ratio of PA images was further demonstrated with a human volunteer study. Our results show that the proposed framework could be valuable in various clinical and preclinical applications to enhance PA image reconstruction.

## 1 Introduction

PA imaging (also called optoacoustic imaging) is a hybrid modality that combines rich optical contrast from optical imaging, with high spatial resolution and large imaging depths from US imaging. In the past two decades, PA imaging has demonstrated great potential for a wide range of applications in preclinical and clinical settings [1], [2], [3]. PA involves the illumination of biological tissues from pulsed or modulated continuous-wave light sources such as solid-state lasers or light-emitting diodes (LEDs). The light is then locally absorbed by endogenous chromophores such as haemoglobin, water and lipids, and exogenous contrast agents. This absorption leads to local temperature increases and transient thermal expansion, serving as a source of US wave generation. The amplitude, frequency, and time-of-flight of the US signal, respectively, provide information on the optical absorption, size and spatial location of the optical absorbers. In PA tomography configurations, the generated US waves propagate through tissue and are then detected with US sensors located on the tissue surface. Subsequently, acoustic inversion is performed on the acquired time series US data to reconstruct PA images representing optical absorption contrast. For this purpose, the SoS in the propagation medium is usually assumed to be homogeneously distributed (typically 1540 m/s as an average for soft tissue). However, the SoS of soft tissue is highly dependent on tissue types, varying from around 1450 m/s (fat) to 1580 m/s (muscle) [4]. Besides, SoS inconsistency can exist between the acoustic coupling medium and the tissue. As such, a constant SoS value of 1540 m/s for the entire propagation medium can lead to significant US aberration artefacts, degrading the image contrast and spatial resolution [5].

Various methods were proposed to mitigate the SoS-induced aberration artefacts in PA tomography [6], [7], [8], [9], [10], [11], [12], [13], [14], [15], [16], [17], [18], [19]. In 2011, Treeby et al. reported an autofocus algorithm to arrive at an optimal single SoS value for the medium by iterative optimisation [6]. The automated selection of the optimal SoS can be achieved by maximising an image resolution metric such as image sharpness [6] and a coherent factor based on delay-compensated US channel data in time domain [7]. These methods implemented a global optimisation of SoS. Image resolution metrics were thus optimised over the whole focus area and local variations on SoS within heterogeneous tissue were neglected. To incorporate the heterogeneity effect, parameterised SoS maps can be directly reconstructed using PA measurements. Several works investigated concurrent recovery of both SoS distributions and initial pressure distributions or optical absorption from PA measurements, which was referred to as a joint reconstruction (JR) problem [9], [10], [11]. Jiang et al. reported a JR approach by seeking numerical solutions to the Helmholtz equation using a finite element method [9]. Zhang et al. proposed a time-domain method based on explicitly exploring two-fold data redundancy in a generalised Radon transform imaging model [10]. Zhang et al. further reported an implicit method by iteratively optimising a cost function for the SoS map and optical absorption simultaneously [11]. Accurate JR may not be achievable due to the numerical instability, as shown in [12], [20]. Therefore, prior information on SoS distributions and detection geometries was usually incorporated for more accurate JR [14], [21]. Cai et al. proposed a feature coupling (FC) based JR method integrated with a full ring array system [18], [19]. The SoS distributions were obtained through an iterative process that maximised the similarity between two PA images reconstructed by the two half-ring data. With recent advances in deep learning (DL), Jeon et al. proposed a DL-based framework for mitigating SoS aberration and streak artefacts resulting from sparse sampling in a linear-array PA imaging system [16]. However, an explicit SoS distribution was not accessible for direct evaluation.

SoS distributions can also be independently measured with US transmission tomography and used for correcting PA image reconstruction in dedicated systems combining US and PA measurements [22]. In adjunction to PA tomography, US tomography can be implemented in a transmission mode by positioning a US transmitter and a receiver in an opposite position [10]. Passive elements can be used as US transmitters in a US transmission tomography configuration based on the PA effect in a PA imaging system by sharing the same US receiver [23], [24]. Mercep et al. proposed a transmission–reflection optoacoustic US (TROPUS) imaging platform that can retrieve multiple acoustic properties including SoS, acoustic attenuation and reflectivity [25]. In such hybrid systems, SoS maps acquired via US transmission tomography are employed for optimising PA image quality during the reconstruction. However, these methods are usually associated with a high degree of complexity, both in hardware design and algorithm development.

Pulse-echo US can be readily integrated with PA imaging by sharing a clinical US array probe for real-time imaging. Such dual-modal imaging systems can provide complementary morphological and molecular information of tissue based on optical absorption. Therefore, this configuration could facilitate the clinical adoption of PA imaging and has thus attracted great attention [26], [27], [28], [29]. Various SoS estimation methods using pulse-echo US signals have been proposed. Gross SoS estimation provides a single averaged SoS between the US transducer face and the focal point [30]. This includes techniques based on the usage of the spatial shift of targets between different angles [31], the calculation of optimal delay profiles of RF data at each channel [32], as well as enhancing B-mode image quality by image deconvolution [33], and evaluation of relevant parameters such as lateral spatial frequency [34], phase variance [35], speckle characteristics [36], [37], and image sharpness [38]. However, the performance of these methods is likely to be affected by SoS inhomogeneities for in vivo applications. Localised SoS estimators have also been developed using pulse-echo acquisition geometries. The techniques include the crossed-beam and beam tracking methods [39], [40], registered virtual detectors [41], and US computational tomography in echo mode (CUTE) [42]. The CUTE method employed a forward model that related the spatial phase shifts of beamformed echoes acquired from different angles to the SoS distribution [42]. Recent extension work demonstrated the improved SoS estimation accuracy and quality using an in vivo human liver model [43], [44]. Besides, model-based SoS reconstruction methods were also proposed as the SoS retrieval from pulse-echo RF data was intrinsically an ill-posed inverse problem [45], [46], [47]. However, limitations arise in the PA/US implementation using a clinical pulse-echo US probe, where constraints on the number of steering angles and retained bandwidths hinder the practicality of most of the SoS estimators. In addition, model-based SoS reconstruction often necessitates prior assumptions and regularisation and optimisation methods to achieve accurate reconstructions. The associated challenges such as convergence issues, high computational demands [44], [47], and algorithmic complexity may limit its applicability.

Recently, there has been growing interest in harnessing DL techniques for SoS estimation using pulse-echo data. Feigin et al. proposed a DL framework for SoS estimation from pulse-echo US RF data acquired from three-plane wave transmits [48]. The DL model was trained on simulated data generated based on simplified tissue models. It demonstrated high estimation accuracy on simulated data but suffered from severe degradation when applied to real data. Similarly, Jush et al. explored the possibility of DL-based SoS estimation using single plane wave transmits [49]. Despite promising results on pure simulated data, DL-based methods faced challenges in generalisation when tested on real data, primarily due to a domain shift between simulation and real-world measurements. In a subsequent study by Jush et al. [50], realistic tissue models, incorporating the intricate structures of breast tissue, were employed to simulate training data. This approach significantly improved the robustness of DL-based SoS estimation on both dissimilar simulated and real data. Moreover, a recent investigation by Simson et al. [51] further supported these findings, indicating that DL-based SoS estimation using simulated data benefited from the utilisation of randomised and anatomically realistic tissue modelling. While utilising simulated data for DL-based SoS estimation offers advantages such as overcoming the limited availability of real data with annotated SoS values, constructing tissue models for US simulation that accurately represent realistic anatomical structures and properties, as well as faithfully simulating the US acquisition system, remain challenging. More importantly, the robustness of the DL model was found to be limited when applied to real data.

In this work, we introduce a novel learning-based approach aimed at retrieving SoS distributions using US radio-frequency (RF) channel data to augment PA image reconstruction in a dual-modal PA/US imaging system employing a clinical probe. Our method involves training a deep neural network to estimate SoS distributions from US RF channel data obtained from digital phantoms via 2D simulations. Further, we improved its performance through transfer learning using data derived from physical phantoms. We evaluated the framework’s performance, encompassing SoS estimation and aberration correction in PA imaging, across diverse scenarios including digital phantoms featuring previously unseen structures and echogenicities, agar-based tissue-mimicking phantoms, ex vivo porcine tissues, and in vivo human vasculature. Consequently, we demonstrate the feasibility of leveraging inherent SoS information within co-registered US data to augment PA reconstruction, circumventing the need for time-intensive iterative algorithms or intricate hardware developments. This work paves the way for the clinical translation of linear array-based PA imaging techniques. Notably, to the best of our knowledge, this study represents the first endeavour to utilise pulse-echo US RF data for enhancing PA image reconstruction, while also exploiting transfer learning techniques for SoS estimation within pulse-echo US imaging.

**Fig. 1:**
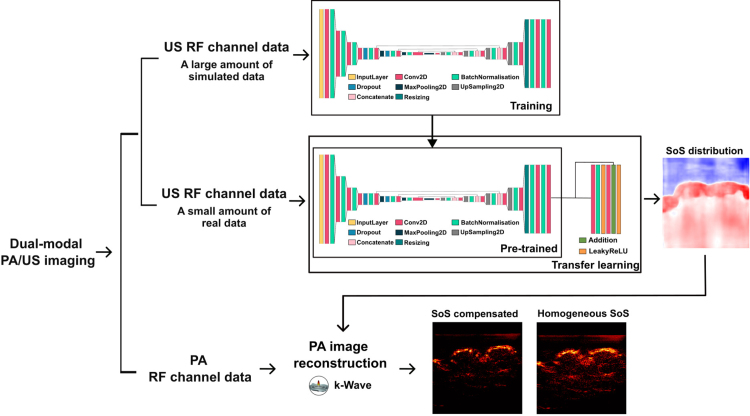
Learning-based sound speed estimation and aberration correction framework for dual-modal photoacoustic (PA)/ultrasound (US) imaging. Top branch: Learning-based speed of sound (SoS) estimation using US radio-frequency (RF) channel data. Bottom branch: SoS compensation for PA image reconstruction (PA images obtained from cross sections of human fingers in vivo).

## 2 Materials and methods

This section is structured as follows: Section 2.1 details the digital simulations used for generating US data for training. Section 1 describes the establishment of the DL model used to estimate SoS distribution from US channel data, including a pre-training stage on simulated data and a transfer learning on small amounts of real data. Section 2.3 introduces a PA image reconstruction method that incorporates DL-based SoS estimation. Section 2.4 details the experimental validation of the DL model including data acquisition and evaluation metrics for qualitative and quantitative analysis.

### 2.1 Ultrasound simulations

To circumvent the lack of a large amount of real US data with explicit SoS distributions, training data was prepared by US simulations in 2D using the k-Wave MATLAB toolbox [52]. A clinical linear array US probe was simulated. The probe had 128 elements spanning 38.4 mm with a pitch size of 0.3 mm and a central frequency of 7 MHz. The dimensions of the simulation grid were 1536 × 1536 with a spatial resolution of 0.025 mm. The probe was located at one side of the grid, with 11 grid points per piezo element and 1 grid point per kerf (the gap between two adjacent elements). The excitation pulses for single-plane wave transmissions were 2-cycle tone burst signals with a central frequency of 7 MHz.

The US data were simulated based on the tissue models of organs and lesions used in [49]. Ellipses representing US heterogeneities with various dimensions and orientations were randomly distributed within a background medium. The dimensions of the medium were 38.4 mm × 38.4 mm. The long axis of the ellipses spanned from 0 mm to (38.4 $\times \sqrt{2}$) mm while the short axis ranged from 0 mm to (19.2 $\times \sqrt{2}$) mm. SoS values were randomly chosen from a uniform distribution $U_{\left[1400,1600\right]}$ for the homogeneous background and from a [1% - 7%] higher range for the inclusions. The echogenicity was considered by simulating the elliptical-shaped inclusions as being hyperechoic, a strategy demonstrated to yield promising generalisation performance across different echogenicity patterns [53]. This was accomplished by elevating the SoS values of the speckles inside the inclusions, enhancing the contrast. Specifically, hyperechoic features were simulated by randomly assigning the SoS values with increments ranging from [7% - 11%] relative to the background at approximately 10% of the grid points. Acoustic attenuation and mass intensity were fixed to 0.5 dB/(MHz cm) and 1020 kg/m^3^ according to the average values of human soft tissues. The speckle density had a mean distribution of 3 speckles per λ^2^ (λ is the wavelength of the transmit pulse). The intensity was assigned by sampling the scatterers with a uniform distribution $U_{\left[-0.03,0.03\right]}$. RF data collected by the US transducer elements were downsampled to achieve a sampling rate of 20 MHz, consistent with the sampling rate of the US imaging facilitated by the clinical probe. This process resulted in US RF data in the form of a 128 × 1024 matrix. A time gain compensation of 0.5 dB/MHz/cm at 1540 m/s was implemented. To eliminate electrical cross-talk noise from the input pulse, the initial 50 samples of RF channel data were zeroed out. Finally, the acquired multichannel RF data were normalised to have a mean of 0 and a standard deviation of 1 for each channel.

Furthermore, thermal noise and system noise were considered with the RF data. Thermal noise resulting from electrons’ agitation in US imaging systems was modelled as white Gaussian noise. The noise amplitude at each channel was determined by the signal-to-noise ratio (SNR) that was randomly sampled from −80 dB to −40 dB. System noise associated with transmission interference was sampled from real US measurements. The system noise was obtained by sampling the signals corresponding to the first 50 out of 1024 samples using real US RF data from in vivo measurements. The US simulations of 6000 samples took around 2 days with an NVIDIA Quadro RTX 5000 GPU.

### 2.2 Deep neural networks for SoS estimation

The deep neural networks Λ were tailored for the simulated US dataset from the networks proposed in [53], [54]. The input and output of Λ were specified as:

(1) $$\Lambda :C_{n\times m}\mapsto S_{p\times q}$$

The US RF channel data C acquired through pulse-echo US with a single plane wave transmission were taken as the input with a size of n×m, where n = 128 is the number of channels and m = 1024 is the number of time steps at a sampling rate of 20 MHz. The output is the corresponding SoS map S with dimensions of 384 × 384 (p×q) and a uniform spatial resolution of 0.1 mm.

As depicted in Fig. 1, the main model was based on a fully convolutional neural network architecture configured as an encoder–decoder. The encoder had six layers, each employing strided convolution to accommodate the non-square input size of 128 × 1024. The strided convolution had non-square kernels starting from a size of 3 × 15. With each convolution layer, the width of the kernel size decreased by 2, resulting in a final kernel size of 3 × 3. The first four layers consisted of strided convolution, followed by LeakyReLU, and Batch Normalisation (BN). Subsequently, the next three layers consisted of strided convolution, LeakyReLU, MaxPooling, and BN. The decoder comprised six layers, with the first five layers employing strided convolution, starting with a kernel size of 3 × 3. In each convolution layer, the width of the kernel size was increased by 2, culminating in a final kernel size of 3 × 11. Analogous to the encoder, the first four layers consisted of strided convolution, LeakyReLU, and BN. The fifth layer consisted of strided convolution and Resizing. The sixth layer consisted of 3 × 3 convolution and BN. The last layer was 1 × 1 convolution. The encoding and decoding paths were interconnected by concatenating the output of the fifth, sixth and seventh encoder layers to the ninth, tenth, and eleventh decoder layers, respectively. The Xavier initialisation was employed for weight initialisation at the convolution layers [55]. The model was trained on 6000 samples with a train/valid split of 0.9. Mean Square Error (MSE) was used as the loss function. Stochastic gradient descent (SGD) with a mini-batch size of 10 and a learning rate of 0.0001 was used for training. After 100 epochs, the network converged to a Root Mean Square Error (RMSE) of 22.90 m/s on the training set and 26.74 m/s on the validation set, respectively.

Real data have characteristics (such as the spatially-variant frequency response and sensitivity of the individual transducer element) that may not be faithfully represented by digital simulations, destructing the performance of the model. Moreover, the model can be sensitive to out-of-plane artefacts and noise, which were not incorporated in the digital simulations. Therefore, a transfer learning strategy of the previously trained model was proposed. As illustrated in Fig. 1, transfer learning was performed with a small amount of real data acquired from agar-based tissue-mimicking phantoms. These phantoms made with agar (Agar powder, Sigma-Aldrich, Germany) and glass beads (0–63μm, Boud Minerals Limited, UK) were prepared following a standardised protocol [56]. The reference SoS value for each agar concentration was measured using an insertion method and used for generating the corresponding SoS distribution for transfer learning (see Supplementary Materials, Sec. 1). The corresponding PA/US RF channel data were obtained with a commercially available LED-based PA/US imaging system (AcousticX, CYBERDYNE INC, Tsukuba, Japan). The base model was augmented by adding a residual block on top of its output to further improve its performance with real data [57]. During the transfer learning, the networks that were pre-trained with a large amount of simulated data were frozen. The parameters of the augmented layers were updated using the MSE as the loss function and SGD. The real dataset consisting of 48 samples was used for training with a train/valid spit of 0.9. The training was stopped after 20 epochs when the MSE was not decreasing on both the train and validation set. All the experiments were conducted on an NVIDIA DGX cluster equipped with 8 A100 GPUs. Model implementations and datasets can be found at: https://github.com/MengjieSHI/learning-based-sos-correction-us-pa.

### 2.3 PA image reconstruction

PA image reconstruction was performed using a time-reversal algorithm implemented in the k-Wave MATLAB toolbox [58]. The computational grid size for PA image reconstruction was 788 (Z)×768 (X) with a uniform spatial resolution of 0.05 mm. The clinical linear array US probe, modelled in the US simulation, was adapted to the simulation grid for PA image reconstruction. The probe was located at one side of the grid (not within the perfectly matched layer (PML) region that absorbed acoustic waves), with 5 grid points per piezo element and 1 grid point per kerf. The size of the PML was set to 10 grid points at both the probe and its opposing edge. The PA RF channel data obtained from real measurements using the LED-based PA/US imaging system had a size of 128 × 1024, which were resampled using an antialiasing lowpass filter to a size of 640 × 4096 and used for the time-reversal reconstruction. The SoS within the simulated medium was specified either as homogeneous or based on DL-based SoS estimation. For the latter, the estimated SoS map with a size of 384 × 384 was interpolated into a size of 768 × 768 pixels and used for defining the acoustic properties of the simulated medium. The acoustic density was 1020 kg/m${}^{3}$. During PA image reconstruction, the first 150 time steps out of 1024 were zeroed out due to the LED-induced system noise. Consequently, a PA image of dimensions 768 × 768 pixels was obtained, featuring a uniform pixel size of 0.05 mm×0.05 mm.

### 2.4 Framework evaluation

The proposed framework was evaluated using data acquired from digital simulations, agar-based tissue-mimicking phantoms, ex vivo tissues, and healthy human volunteers, respectively.

**Fig. 2:**
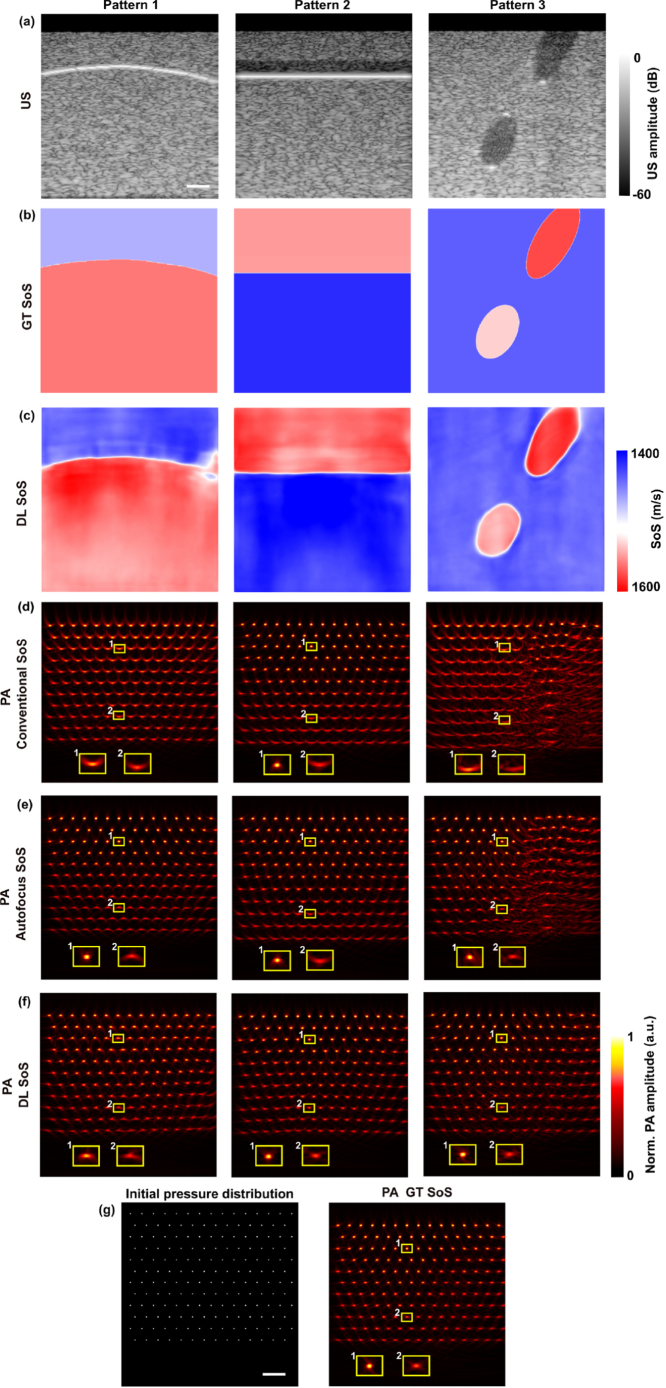
Learning-based sound speed estimation and aberration correction in dual-modal photoacoustic (PA)/ultrasound (US) imaging demonstrated with digital phantoms with point optical absorbers. (a) US B-mode images with the top 2 mm zeroed out due to noise interference. (b) Ground truth of speed of sound (SoS). (c) Deep learning (DL)-based SoS estimation. (d-f) PA image reconstruction using a conventional SoS of 1540 m/s, an autofocus method, and a DL method. (g) Initial pressure distribution used for PA simulation and the corresponding PA image reconstruction. Inserts depict enlarged PA images of point sources for comparison. a.u.: arbitrary unit; Scale bar: 5 mm.

The digital phantoms encompassed diverse anatomical structures and echogenicities not represented in the training data. These included layered structures featuring both straight and deformed boundaries, alongside randomised SoS values and echogenicities, as shown in Fig. 2(a). A total of 120 samples were generated with 40 samples corresponding to each pattern as illustrated in Fig. 2. Point sources were delineated within PA simulations by assigning a constant initial pressure of 1 to 2D grid coordinates. PA data acquisition adhered to the same acoustic forward model defined for PA image reconstruction in Section 2.3.

The model was then tested on real data acquired with agar-based tissue-mimicking phantoms, needle (20G, BD, USA) insertions into ex vivo porcine tissues simulating minimally invasive procedures, and in vivo human vasculature. The tissue-mimicking phantoms were composed of two layers of agar phantoms with the same or different concentrations of agar and glass beads (2% w/v, 4% w/v, and 6% w/v for agar; 1% w/v and 0.5% w/v for glass beads). Three pencil leads (Faber-Castell, Stein, Germany) with a diameter of 0.5 mm were randomly positioned in the phantoms as optical absorbers. With ex vivo experiments, SoS aberration artefacts around the needle tip were demonstrated with out-of-plane and in-plane insertions, respectively. For the out-of-plane insertion, the PA signals of the needle tip were enhanced at deep depths by integrating a fibre-optic US transmitter within the needle lumen [27]. Furthermore, the framework was tested using in vivo 3D human vasculature by scanning the wrist of a healthy volunteer. The human volunteer experiments were approved by the King’s College London Research Ethics Committee (study reference: HR-18/19-8881). The probe scanned over a length of 4 cm on a human wrist using a linear translation stage. A total of 1536 frames were obtained. Frame averaging was implemented across 4 consecutive frames to improve the SNR, resulting in 384 frames for model evaluation. For all experiments, water served as the acoustic coupling medium. Prior to and following imaging procedures, the temperature of the water was meticulously measured to facilitate the determination of the SoS.

The performance of SoS estimation and subsequent aberration correction using PA image reconstruction was compared with the autofocus method [6] and conventional assumption of 1540 m/s. For detailed implementation specifics of the autofocus method, please refer to Supplementary Materials, Sec. 2.

Root mean square error (RMSE) was employed to quantify the accuracy of SoS estimation. With a SoS map SoS${}_{est}$ and its corresponding ground truth SoS${}_{gt}$ of size p×q, the RMSE was given by:

(2) $$RMSE=\sqrt{\frac{1}{pq}\overset{p}{\underset{i=1}{\sum }}\overset{q}{\underset{j=1}{\sum }}{\left[{\mathrm{SoS}}_{est}\left(i,j\right)-{\mathrm{SoS}}_{gt}\left(i,j\right)\right]}^{2}}$$

For the digital phantoms, the RMSE was calculated within the structure of the same SoS and across the propagation medium. SoS${}_{est}$ was a homogeneous distribution for the autofocus method or using the conventional assumption of 1540 m/s. For agar-based tissue-mimicking phantoms, the RMSE of DL-based SoS estimation was calculated for different media, including water, agar 2%, agar 4%, and agar 6%. A total of 60 measurements were utilised for statistical analysis, comprising 20 measurements from each concentration. Additionally, the local structural similarity index (SSIM) served as the evaluation metric for aberration correction evaluation during PA image reconstruction, as calculated by:

(3) $$\mathrm{SSIM}=\frac{\left(2\mu_{x}\mu_{y}+C_{1}\right)\left(2\sigma_{xy}+C_{2}\right)}{\left(\mu_{x}^{2}+\mu_{y}^{2}+C_{1}\right)\left(\sigma_{x}^{2}+\sigma_{y}^{2}+C_{2}\right)}$$

where $\mu_{x}$, $\mu_{y}$, $\sigma_{x}$, $\sigma_{y}$, $\sigma_{xy}$ represent the means, standard deviations, and the cross-variance of reconstructed PA images with aberration correction using DL-based SoS and the corresponding reference SoS, respectively. $C_{1}$ and $C_{2}$ were calculated by $C_{1}=0.01L^{2}$ and $C_{2}=0.03L^{2}$, respectively where L is set to 1 based on the dynamic range of PA pixel intensity. For the digital phantoms, the SSIM was calculated within the structure of the same SoS and across the propagation medium. PA images reconstructed using the correct SoS served as the reference. For the agar-based tissue-mimicking phantoms, the measured SoS values of water and agar phantoms were used for acquiring reference PA image reconstruction. Furthermore, lateral profiles were plotted along the local maxima corresponding to optical point sources (pencil leads). The Full Width at Half Maximum (FWHM) values extracted from these profiles provided insights into the reconstructed dimensions of the pencil leads.

For in vivo human data, the enhancement achieved by aberration correction in PA image reconstruction was evaluated using SNR. The SNR was calculated by:

(4) $$SNR=20\log_{10}\frac{\mu }{\sigma }$$

where μ is the mean amplitude of tissue signals, σ is the standard deviation of background noise. The pixels with an intensity below 0.35 were classified as background noise, while those with an intensity of 0.35 or higher were classified as tissue signals.

## 3 Results

### 3.1 Digital phantoms

Fig. 2 shows three representative results from digital phantoms. Pattern 1 features a 2-layer structure with a curved boundary and a uniform echogenicity distribution. The DL model was able to detect SoS discrepancy between two layers, disregarding their identical speckle contrast, with RMSE of 38.37 m/s and 17.63 m/s for each respective layer. In contrast, the global SoS optimisation regarding the image sharpness of PA reconstruction by the autofocus method [6] resulted in RMSE of 6.23 m/s and 77.72 m/s for each layer, respectively. The SoS estimation results by the autofocus method are presented in Fig.s3 in Supplementary Materials, Sec. 2. On a global scale, the DL method achieved the lowest RMSE of 25.68 m/s outperforming both the conventional SoS of 1540 m/s (RMSE: 40.39 m/s) and the autofocus method (RMSE: 65.11 m/s). Additionally, SSIM was employed for evaluating the performance of aberration correction in PA imaging using the aforementioned SoS estimation. The DL method demonstrated comparable performance to the autofocus method, with SSIM values of 0.77 and 0.78, respectively. Reconstructed PA images are shown in Fig. 2(d–f). Aberration artefacts were apparent in the PA image reconstructed under the conventional SoS of 1540 m/s. While the autofocus method corrected the artefacts at layer 1, it failed at layer 2 due to the incorrect SoS estimation. In contrast, the DL method achieved robust aberration correction using the estimated SoS map. It is noteworthy that the performance of aberration correction with the DL method degraded at deep depths (≥ 3 cm). This could be attributed to the SoS estimation errors near the tissue boundary, which was probably due to the presence of the reflection artefacts.

**Table 1:** Comparison of root mean square error (RMSE) for speed of sound (SoS) estimation and structure similarity index measure (SSIM) for aberration correction using conventional assumption, autofocus, and deep learning (DL) methods. Best results obtained for each pattern were highlighted.

		RMSE (m/s)			SSIM		
		Conventional	Autofocus	DL	Conventional	Autofocus	DL
Pattern 1	Layer 1	71.08	6.23	38.37	0.63	0.94	0.78
	Layer 2	12.84	77.72	17.63	0.68	0.66	0.78
	Global	40.39	65.11	**25.68**	0.66	**0.78**	0.77

Pattern 2	Layer 1	1.27	17.80	22.44	0.88	0.83	0.82
	Layer 2	124	102.86	12.75	0.63	0.68	0.81
	Global	99.90	85.22	**18.14**	0.76	0.76	**0.81**

Pattern 3	Background	103.87	11.43	9.18	0.54	0.73	0.87
	Inclusions	27.55	126.18	15.67	0.50	0.54	0.82
	Global	97.69	45.76	**10.21**	0.51	0.69	**0.86**

**Table 2:** Statistical analysis of speed of sound estimation using deep learning. N: number of samples.

	Pattern 1 (N = 40)	Pattern 2 (N = 40)	Pattern 3 (N = 40)
RMSE (m/s)	27.95 ± 8.61	32.25 ± 7.83	12.18 ± 3.74

Pattern 2 depicts structures in US images with straight surfaces and isoechoic layers. Despite the echogenicity varying in the top two layers, the SoS prediction remained consistent using the DL method, with an RMSE of 22.44 m/s. For the bottom layer (Layer 2 in Table 1), the DL method yielded a much smaller RMSE of 12.75 m/s compared to 102.86 m/s obtained by the autofocus method and 124 m/s by the conventional assumption. Thus, PA image reconstruction was enhanced by incorporating the DL-based SoS estimation, as shown in Fig. 2(e), middle column, with an SSIM of 0.81 compared to 0.76 by both the autofocus method and the conventional assumption.

Besides, Pattern 3 mimics a specfic type of tissue anatomical structures in US images (e.g. breast abnormalities), featuring a homogeneous background and hypoechoic elliptical inclusions. The DL model effectively retrieved the SoS distributions of both the background and the inclusions, achieving RMSEs as small as 9.18 m/s and 15.67 m/s for the background and inclusions, respectively. The enhancement in PA image reconstruction was also observed as shown in Fig. 2(e), right column. However, the autofocus method introduced large SoS estimation errors (up to 126.18 m/s in RMSE for the inclusions), as evidenced by the severe distortions in the PA image (Fig. 2(d) right column). Similar results were observed with the conventional assumption. Furthermore, the generalisation performance of the DL model was statistically analysed using a total of 120 samples (40 samples randomly selected from each representative pattern) and summarised in Table 2. The achieved RMSEs were close to those acquired during the model training, with the smallest RMSE of 12.18 m/s observed in the group with US anatomy assembled to Pattern 3.

### 3.2 Agar-based tissue-mimicking phantoms

**Fig. 3:**
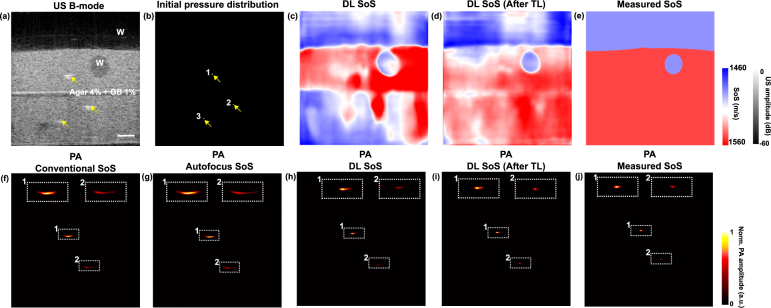
Learning-based sound speed estimation and aberration correction in dual-modal photoacoustic (PA)/ultrasound (US) imaging demonstrated with agar-based tissue-mimicking phantoms. (a) US B-mode image. (b) Initial pressure distribution (c) Deep learning (DL)-based SoS estimation. (d) DL-based SoS estimation after transfer learning (TL). (e) Measured SoS. (f-j) PA image reconstruction using a conventional SoS of 1540 m/s, an autofocus method, DL methods, and measured SoS. The presence of water (W) is indicated. Point sources (pencil leads) are denoted by yellow arrows. Inserts depict enlarged PA images of point sources for comparison. a.u.: arbitrary unit; Scale bar: 5 mm.

An exemplar result is shown in Fig. 3. As shown in the B-mode image, the phantom consisted of two layers made with the same agar concentrations of 4%. A tubular structure with a diameter of around 5 mm was introduced at the upper layer. Meanwhile, three pencil leads serving as point sources appeared in the B-mode image with a higher speckle intensity than the background, as indicated by the yellow arrows.

**Table 3:** Statistical analysis of DL-based SoS estimation using agar-based tissue-mimicking phantoms. The total number of samples was 60 with 20 samples per each agar concentration. TL: transfer learning.

RMSE (m/s)	Before TL	After TL
Water	44.2 ± 22.1	**15.5 ± 1.5**
Agar 2%	40.4 ± 19.3	**26.7 ± 7.7**
Agar 4%	41.9 ± 20.4	**16.0 ± 1.1**
Agar 6%	37.7 ± 16.7	**15.2 ± 5.7**

Fig. 3(f–j) compared the PA images reconstructed using different SoS estimation methods. The conventional SoS assumption of 1540 m/s distorted the cross-sectional PA signals of the pencil leads. For point source 1, the lateral FWHM was 1.52 mm compared to the actual dimension of 0.50 mm (lateral resolution of the imaging system: 0.59 mm [29]). For point source 2 at a deeper depth, the aberration was accumulated, resulting in a lateral FWHM of 2.47 mm. The autofocus method yielded a gross SoS of 1560 m/s, underscoring its intrinsic inefficacy for the PA images that contained limited-viewed artefacts [6]. Details of the SoS selection process using the autofocus method are provided in Fig. s4, Supplementary Materials. The PA signals were then reconstructed with the SoS parameterised using the DL output. With the SoS map predicted by the base model (without transfer training using phantom data), the lateral FWHMs of points 1 and 2 were 1.13 mm and 1.29 mm, respectively. However, the performance was degraded at deep depths, especially for the bottom layer, with an average SoS of 1477.5 m/s compared to the measured 1520.3 m/s. In contrast, the updated model demonstrated good performance of SoS prediction at deep depths. The average SoS values for the coupling layer, upper layer, and bottom layer were 1484.4 m/s, 1521.8 m/s, and 1528 m/s, respectively. The lateral resolution, represented by the lateral FWHMs, was improved to 0.97 mm and 0.74 mm for points 1 and 2, respectively. Furthermore, local SSIM based on the regions of interest (ROIs) that enclosed the point sources was calculated using the initial pressure distribution of the phantom for reference, as shown in Fig. 3(d). The point sources were segmented from the US B-mode image, with each having a diameter of 0.5 mm and an initial pressure of 1. Note that the deepest point source was excluded from SSIM evaluation due to its poor SNR resulting from significant light and acoustic attenuation. The SSIM values were 0.78, 0.76, and 0.88 for the PA images reconstructed using the conventional SoS assumption of 1540 m/s, the autofocus method, and the DL SoS estimation after transfer training. Further, a reference SoS map was obtained using the measured SoS values for water (1490 m/s) and agar phantoms (1520.3 m/s), as shown in Fig. 3(j). The corresponding SoS-compensated PA image yielded an SSIM of 0.92 with the initial pressure image as a reference. However, it is important to note that the anatomical segmentation for SoS parameterisation at the boundaries and tubular inclusions may generate errors due to the poor US contrast.

The model was evaluated using different agar-based tissue-mimicking phantoms. Additional results were included in Supplementary Materials, Fig. s4. Table 3 compared the estimation errors of SoS for different media including the coupling water and tissue-mimicking phantoms containing different agar concentrations.

**Fig. 4:**
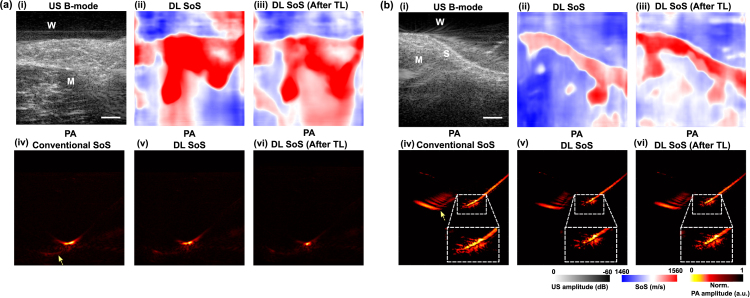
Learning-based sound speed estimation and aberration correction in dual-modal photoacoustic (PA)/ultrasound (US) imaging demonstrated with clinical needle in-plane (a) and out-of-plane insertions into porcine tissues ex vivo (b). (i) US B-mode images. (ii–iii) Deep learning (DL)-based speed of sound (SoS) estimation. DL SoS: SoS estimation using the DL model trained solely on simulated data. DL SoS (After TL): SoS estimation using the DL model after transfer learning (TL) on physical phantom data. (iv) PA image reconstruction using a homogeneous SoS of 1540 m/s (iv–vi) PA image reconstruction using DL-based SoS estimation before and after TL. Yellow arrows denote image artefacts associated with acoustic reflection and limited view. a.u.: arbitrary unit; M: Muscle tissue; S: skin. W: water. Scale bar: 5 mm.

### 3.3 Ex vivo tissue experiments simulating interventional procedures

Acoustic aberration can distort the PA visualisation of medical devices, such as clinical metallic needles that are commonly used during PA/US-guided minimally invasive procedures [29]. In Fig. 4(a), a clinical spinal needle with enhanced tip visualisation [27] was inserted into muscle tissues using an out-of-plane approach. The tip signals in the PA reconstructions were distorted with a conventional homogeneous SoS of 1540 m/s; for the in-plane needle insertion in Fig. 4(b), the distal segment of the needle experienced a bending distortion (shown in the zoom-in images). Both of them led to ambiguity in tip localisation under PA guidance. The estimated SoS of water was 1497.7 m/s, matching the literature value at 20 °C (1490 m/s). Besides, the SoS for muscle tissues and the compound layer of skin and fat in the joint tissue were estimated as 1547.6 m/s and 1558.6 m/s, respectively. The estimations were acceptable, given the known variation in SoS, ranging from 1436 m/s to 1470 m/s for the fat layers and 1682 m/s for skin [59]. By employing the US-informed SoS compensation for PA reconstruction, the aberration artefacts at the tip area were suppressed, as shown in Fig. 4(v–vi). Additionally, the DL model exhibited enhanced SoS estimation performance following transfer learning compared to its performance when trained solely on simulated data, as shown in Fig. 4(vi).

### 3.4 In vivo human data

**Fig. 5:**
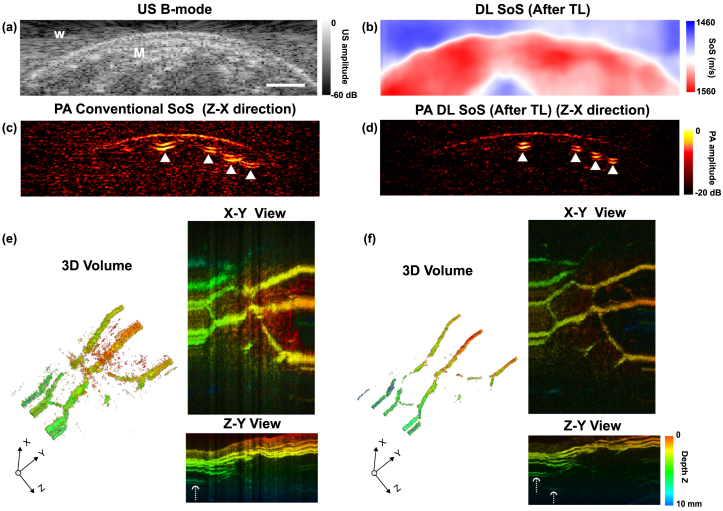
Learning-based sound speed estimation and aberration correction in dual-modal photoacoustic (PA)/ultrasound (US) imaging demonstrated with in vivo 3D volume of the human wrist. (a) US B-mode image. (b) SoS estimation using the DL model after transfer learning (TL) on physical phantom data. (c) PA image reconstruction using a conventional SoS of 1540 m/s. (d) PA image reconstruction using DL-based SoS estimation after TL. (e) 3D volumes and maximum intensity projections from X-Y and Z-Y directions. Cross-sectional blood vessels are denoted by white triangles. Longitudinal sections of blood vessels are denoted by white arrows. W: water, M: muscle. Scale bar: 5 mm.

A 3D scanning sequence consisting of 384 2D imaging slices of a human wrist was used to evaluate the model’s performance, focusing on its reproducibility on the data of low SNRs. The recovered SoS value of the muscle tissue by the DL method was 1546.0 m/s ± 6.0 m/s, well corresponded to the known SoS of human soft tissues (1540 m/s) [4]. The estimated SoS of water was 1502.7 m/s ± 4.2 m/s. Fig. 5(c) and (e) show the PA images reconstructed with a conventional SoS value of 1540 m/s. The cross-sections of blood vessels, denoted by white triangles, exhibited substantial aberration artefacts. In contrast, the PA images incorporating DL-based SoS compensation presented enhanced visualisation of blood vessels, with an SNR improvement from 17.9 dB ± 0.7 to 20.4 dB ± 0.6 (p<0.001). PA images reconstructed using a homogeneous SoS of 1500 m/s (measured water SoS) are shown in Fig. s6, Supplementary Materials. The autofocus method failed to determine the optimal SoS, as shown in Fig. s7, Supplementary Materials. The enhancement can be further observed in the depth-coded 3D reconstructions and the corresponding maximum intensity projections from X-Y and Z-Y directions, respectively (shown in Fig. 5(e–f)). The DL-based aberration correction can be potentially helpful for visualising small vessels, particularly those susceptible to noise contamination in PA signals, or vessels at deep depths where PA signals were diminished due to significant light attenuation (denoted by white arrows in Fig. 5(e–f) Z-Y view).

## 4 Discussion

A long-standing challenge in PA image reconstruction is the lack of accurate information on SoS variations in heterogeneous biological tissue. Failure to compensate for the variation in SoS can result in severe image distortions and artefacts, which compromise the image quality. Prior studies have explored different approaches to incorporate the SoS heterogeneity during PA image reconstruction. Although promising results have been reported, they usually involve certain complexities such as dedicated imaging hardware and sophisticated algorithm developments. Recent studies have showcased the ability of DL to estimate SoS distributions from raw US RF channel data in pulse-echo US using simulated data [49], [53], [54], [60]. However, DL-based SoS estimators have faced challenges for practical applications, primarily attributed to the domain shift between simulation and real measurements. While large, heterogeneous, and anatomically realistic simulated datasets hold promise for DL-based SoS estimation, their generation remains non-trivial. In this work, training data were generated using a US simulation pipeline established using simplified tissue models featuring elliptical inclusions representing organs and lesions. Experimental findings demonstrated the DL model’s ability to generalise across dissimilar anatomical structures using digital phantoms. Notably, with transfer learning, the DL model exhibited robust generalisation performance on real data, indicating its potential for clinical and preclinical applications.

The dual-modal system can acquire interleaved PA and US data for real-time applications, making it possible to enhance PA image reconstruction using SoS information inherited in the corresponding co-registered US data. The dataset for training a fully convolutional deep neural network was prepared in silico in 2D, taking into consideration the variations of US anatomy and SoS values as well as computational efficiency. Noise including thermal noise and system noise from real measurements was added. Even though the digital phantoms used for model evaluation contain anatomical structures and echogenicity patterns that differ from the training distribution, the trained network can estimate their SoS distributions with high fidelity. The estimated SoS maps can compensate for whole-field SoS variations during PA reconstruction. In comparison, conventional and an autofocus method [6] based on maximising image sharpness resulted in deformations in point source reconstructions as evidenced by degradation in the lateral resolution.

The trained network had a degraded performance when applied to measurement data, which could be attributed to its poor robustness against out-of-plane artefacts and system noise. It was observed that transfer learning using even a small amount of data acquired from physical phantoms was able to improve the model performance. The estimated SoS values for soft tissues were situated in the range of the values reported in the literature and were found to be effective in mitigating the SoS aberration artefacts in the conventional PA reconstruction. In particular, the network can identify the SoS inconsistency between coupling medium (water) and soft tissues, which was of importance to correct the positions of medical devices such as metallic needles. This could be advantageous for tracking the needle tip relative to the patient anatomy during various US-guided minimally invasive procedures such as peripheral nerve blocks and tumour biopsy [27], [61], [62], [63]. Also, the network after transfer training generalised well when applied to in vivo human data. The averaged SNR of the imaged vasculature improved from 17.9 dB to 20.4 dB using the proposed DL-based aberration correction method. This is particularly useful for LED-based systems where small capillaries can be readily suppressed by thermal noise due to the low optical fluence. Moreover, limited-view artefacts represent a significant source of image degradation in linear array-based PA/US imaging. Additional approaches aimed at mitigating limited-view artefacts, such as model-based techniques [64] and DL-based methods, can be incorporated to further enhance PA image quality [65].

The network took around 0.01 s for a single frame SoS estimation with a GPU (Tesla T4), indicating the potential for real-time PA image reconstruction with SoS compensation. The current implementation of time reversal-based PA image reconstruction in k-Wave required approximately 1 min for a computational grid size of 788 × 768 when using an NVIDIA Quadro RTX 5000 GPU. It is important to note that computational time is contingent upon the total size of the simulation grid including the PML. Future works could include the development of a fast image reconstruction algorithm that incorporates SoS distributions in tissue such as a deep neural network [64], [66].

The proposed learning-based aberration correction method establishes a foundational framework for dual-modal PA/US imaging systems sharing clinical US probes. Looking ahead, the DL model can serve as a pre-trained model and be fine-tuned on small datasets that resemble characterised tissue structures such as layered structures for skin pathology, to further enhance its applicability and performance. Future endeavours could also include methods for improving the performance of DL-based SoS estimation in pulse-echo US such as coherency input [67], [68] and diverse domain adaptation techniques [69] in comparison with existing network architectures and datasets. Furthermore, given the valuable insights into pathology diagnosis provided by SoS in tissue, further studies are required to assess the accuracy of SoS estimation in patients towards clinical translation.

## 5 Conclusions

We developed a DL framework for accurate PA image reconstruction with SoS compensation on a dual-modal PA/US imaging system exploiting a clinical US probe, which is particularly suitable for real-time applications. This framework utilises US RF channel data to estimate SoS distributions and correct aberration artefacts in PA images resulting from SoS heterogeneity. By employing transfer learning with agar-based tissue-mimicking phantoms, the DL model demonstrated improved generalisation and robustness to experimental ex vivo and in vivo data. Thus, this framework holds potential for preclinical and clinical applications, including the facilitation of image-guided minimally invasive medical procedures.

## CRediT authorship contribution statement

**Mengjie Shi:** Writing – review & editing, Writing – original draft, Visualization, Validation, Software, Methodology, Investigation, Formal analysis, Data curation, Conceptualization. **Tom Vercauteren:** Writing – review & editing, Supervision, Resources, Project administration. **Wenfeng Xia:** Writing – review & editing, Supervision, Resources, Methodology, Investigation, Funding acquisition, Data curation, Conceptualization, Project administration.

## Declaration of competing interest

The authors declare that they have no known competing financial interests or personal relationships that could have appeared to influence the work reported in this paper. T.V. is co-founder and shareholder of Hypervision Surgical Ltd, London, UK.

## Data Availability

Data and codes can be found at: https://github.com/MengjieSHI/learning-based-sos-correction-us-pa.

## Appendix A Supplementary data

MMC S1 Supplementary Material: Experiments on sound speed measurement and additional results and analyses.
