# Supplementary material for: Learning-based sound speed estimation and aberration correction for linear-array photoacoustic imaging
Source: Photoacoustics. 2024 May 28;38:100621. doi: 10.1016/j.pacs.2024.100621 (PMC11637060; doi:10.1016/j.pacs.2024.100621)
Supplement: MMC S1 — Supplementary Material: Experiments on sound speed measurement and additional results and analyses. [file mmc1.pdf]

Supplementary materials for ”Learning-based sound speed estimation and aberration correction for linear-array photoacoustic imaging”

Mengjie Shi[a], Tom Vercauteren[a], Wenfeng Xia[a,*]

[a]School of Biomedical Engineering and Imaging Sciences, King's College London, London, SE1 7EH, United Kingdom

1. Transfer training

1.1. Speed of Sound measurement

The pulse-echo technique was used to measure the speed of sound (SoS) of the agar phantoms. A single-element 3.5 MHz transducer (V382-SU, Olympus, Shinjuku, Japan) was operated in pulse-echo mode at a repetition rate of 1 kHz. The transducer, agar samples, and metal plate were positioned as illustrated in Fig. s1. An ultrasonic pulser/receiver (DPR300, JSR Ultrasonics, Pittsford, USA) was utilised to trigger the transducer as well as capture the echo signals from the metal plate. The signals were analysed using an oscilloscope (DSOX3022G, Keysight Technologies, US) and subsequently saved for offline processing. The SoS of the agar phantoms were calculated using Eq. (1)

$$c_e = \frac{d}{\Delta t + \frac{d}{c_w}} \tag{1}$$

where c_w is the SoS of water at 20.2°C (1490 m/s) [1]. Δt is determined by calculating the cross-correlation between the echo signals acquired with the presence of the agar samples and without them. The reference SoS for 2%, 4%, and 6% agar phantoms at a temperature of 20°C was 1507.1±11.4 m/s, 1511.0±7.4 m/s, and 1520.3±5.6 m/s, respectively.

*Corresponding author

Email address: wenfeng.xia@kcl.ac.uk (Wenfeng Xia)

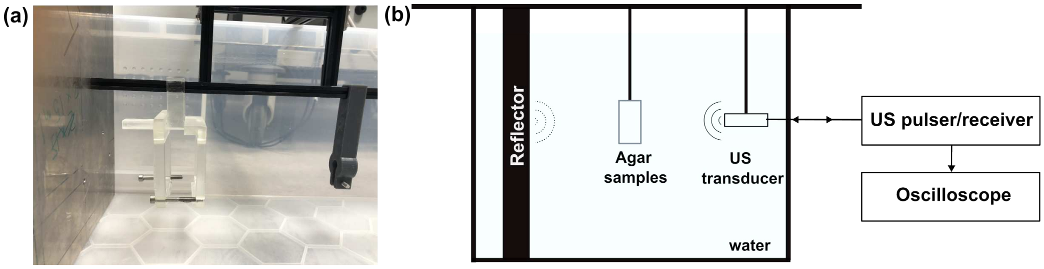

Fig.s 1: Photograph (left) and schematic (right) of the setup for speed of sound measurement.

1.2. Dataset information

A total of 6 permutations were prepared by considering three concentrations of agar (2%, 4%, and 6%) with two concentrations of glass beads (0.5% and 1%). First, the phantom samples were imaged ultrasonically using AcousticX. Then, the phantom samples with the same concentrations, cut into various thicknesses, were randomly stacked to form layer structures. Tubular inclusions, varying in size and filled with water, were incorporated. A few exemplars are shown in Fig. s2. The corresponding US B-mode images were utilised for SoS annotation. SoS parameterisation was based on a normal distribution where the mean was determined by the average of SoS measurements (for each concentration) while the standard deviation was 8 m/s. For the data containing a coupling water layer, a SoS of 1490 m/s was used for the reconstruction. The dataset used for model evaluation was acquired independently. Phantom samples with different concentrations and geometries were assembled to generate the layer structures, differing from the training dataset.

2. Autofocus method

The autofocus approach was implemented based on an example in k-Wave `http://www.k-wave.org/documentation/example_pr_2D_tr_autofocus.php`. In the example, three focus functions including the Brenner gradient, the Tenenbaum gradient, and the normalised variance, were used for estimating image sharpness. The focus function results against different SoS values are shown in Fig. s3. The optimal SoS value for each pattern was determined when it maximised the metrics.

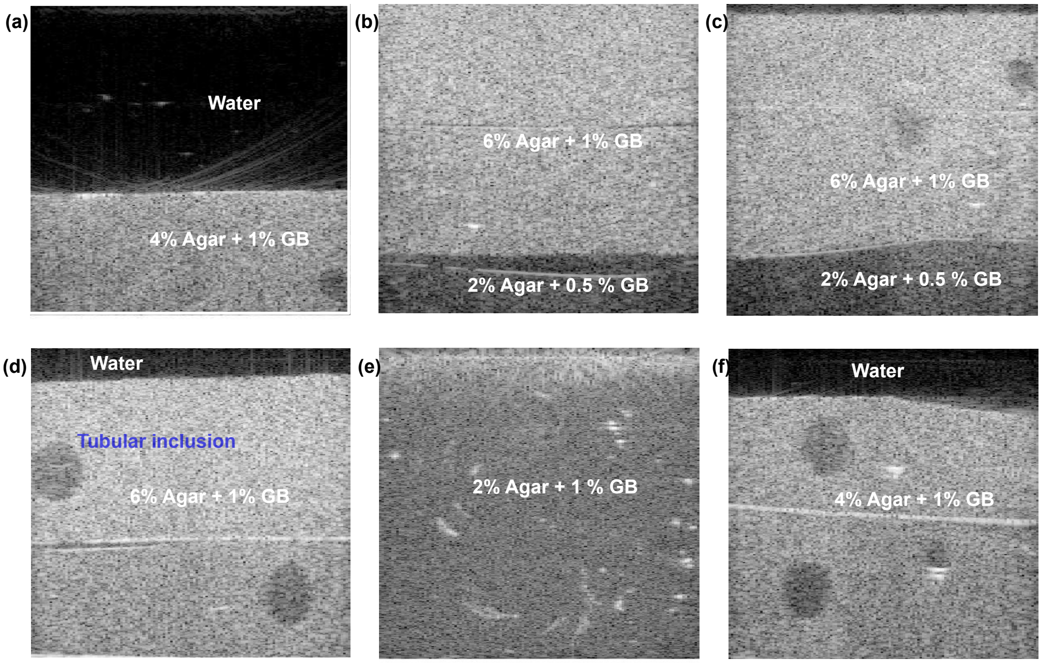

Fig.s 2: US B-mode images of agar-based tissue-mimicking phantoms for transfer training

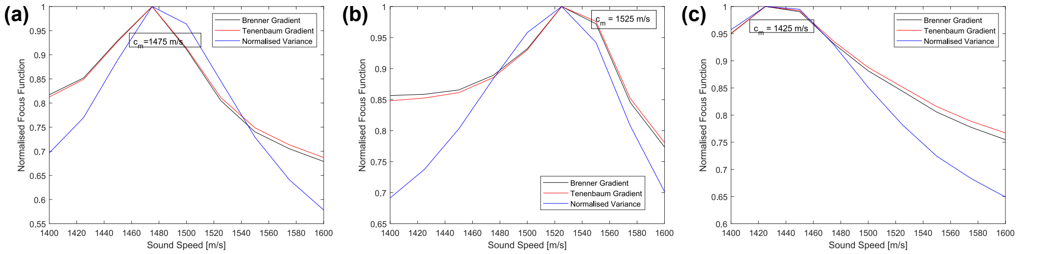

Fig.s 3: Focus functions against different speed-of-sound values for digital phantoms. (a-c) correspond to patterns 1-3 in Fig. 2, respectively

3. Model evaluation

The model was tested using phantom data acquired by imaging agar-based tissue-mimicking phantoms (exemplars shown in Fig. s5). Fig. s5 also compares the model performance before and after transfer training (denoted by Updated DL SoS). The model's performance can be affected by reflection artefacts, as indicated in the last column (SoS estimation at the water layer).

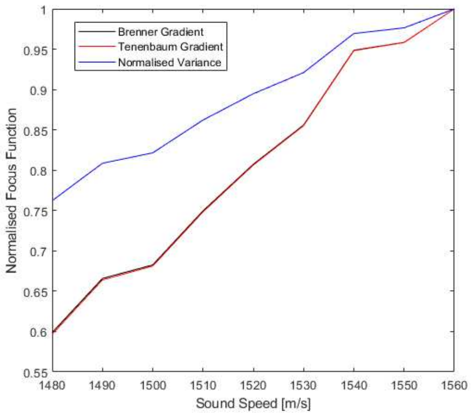

Fig.s 4: Focus functions against different speed-of-sound values for agar-based tissue-mimicking phantoms.

References

[1] J. Mamou, M. L. Oelze, Quantitative ultrasound in soft tissues, Springer, 2013.

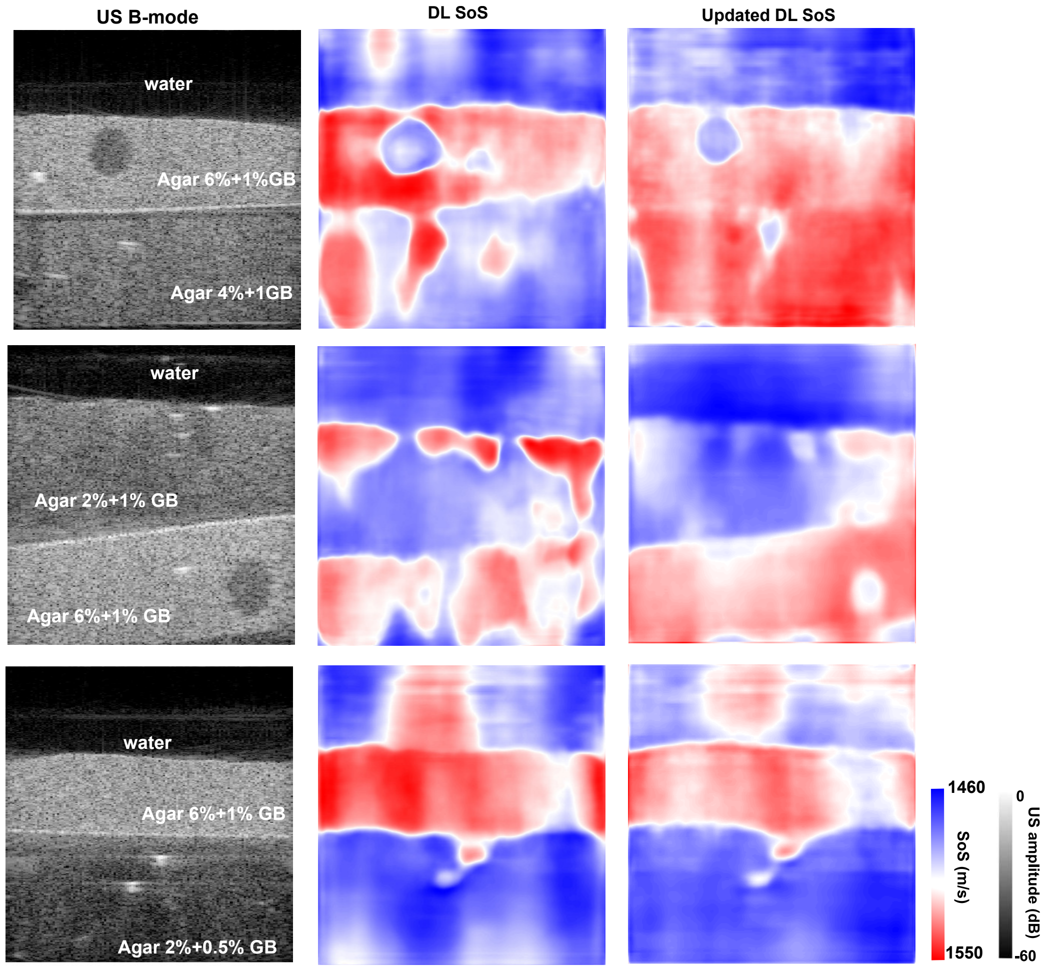

Fig.s 5: Model evaluation using agar-based tissue-mimicking phantoms

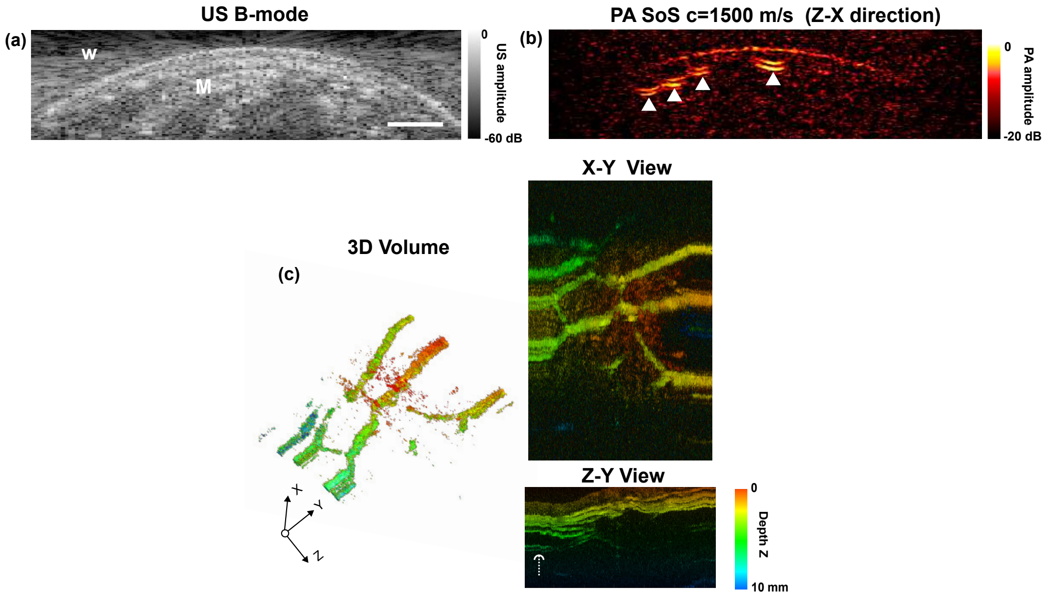

Fig.s 6: Model evaluation using in vivo human data with a homogeneous SoS of 1500 m/s.

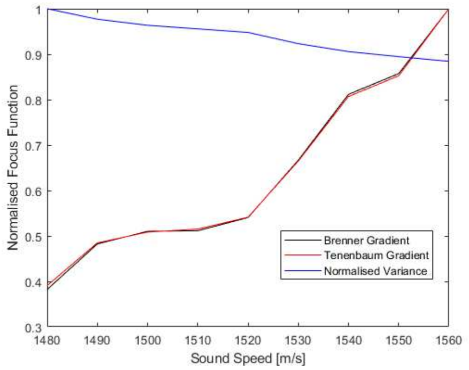

Fig.s 7: Focus functions against different speed-of-sound values for in vivo human data.
